# Laryngeal Features Are Phonetically Abstract: Mismatch Negativity Evidence from Arabic, English, and Russian

**DOI:** 10.3389/fpsyg.2017.00746

**Published:** 2017-05-15

**Authors:** Kevin T. Schluter, Stephen Politzer-Ahles, Meera Al Kaabi, Diogo Almeida

**Affiliations:** ^1^Division of Science, New York University Abu DhabiAbu Dhabi, United Arab Emirates; ^2^Faculty of Linguistics, Philology, and Phonetics, University of OxfordOxford, UK; ^3^NYUAD InstituteNew York University Abu Dhabi, Abu Dhabi, United Arab Emirates; ^4^Department of Chinese and Bilingual Studies, The Hong Kong Polytechnic UniversityKowloon, Hong Kong; ^5^Department of Applied Language Sciences, United Arab Emirates UniversityAl-Ain, United Arab Emirates

**Keywords:** mismatch negativity, laryngeal state, voicing, spread glottis, aspiration, phonological feature, distinctive feature, phoneme

## Abstract

Many theories of phonology assume that the sound structure of language is made up of *distinctive features*, but there is considerable debate about how much articulatory detail distinctive features encode in long-term memory. Laryngeal features such as *voicing* provide a unique window into this question: while many languages have two-way contrasts that can be given a simple binary feature account [±VOICE], the precise articulatory details underlying these contrasts can vary significantly across languages. Here, we investigate a series of two-way voicing contrasts in English, Arabic, and Russian, three languages that implement their voicing contrasts very differently at the articulatory-phonetic level. In three event-related potential experiments contrasting English, Arabic, and Russian fricatives along with Russian stops, we observe a consistent pattern of asymmetric mismatch negativity (MMN) effects that is compatible with an articulatorily abstract and cross-linguistically uniform way of marking two-way voicing contrasts, as opposed to an articulatorily precise and cross-linguistically diverse way of encoding them. Regardless of whether a language is theorized to encode [VOICE] over [SPREAD GLOTTIS], the data is consistent with a universal marking of the [SPREAD GLOTTIS] feature.

## Introduction

The way speech sounds are categorized and stored in long-term memory has long been a central topic of investigation in language research. This line of inquiry has drawn on insights from many different sources, including detailed analyses of the structure of sound patterns of languages (Jakobson et al., 1951; Halle, 1959; Chomsky and Halle, 1968), data pertaining to speech perception and sound categorization (Repp, 1984) and, more recently, neurophysiological evidence (Dehaene-Lambertz, 1997; Phillips et al., 2000; Eulitz and Lahiri, 2004; Mesgarani et al., 2014).

Many theoretical (phonological) models of sound structures of languages have long held that not only are speech sounds organized into discrete phonemic categories, such as the ones represented by the symbols /s/ and /z/, but also that these categories are not atomic (cf. Baković, 2014 for an overview). Instead, sub-phonemic bits of information often termed *distinctive features* are recognized as the elemental components of linguistic sound categories. Here, we assume these distinctive features are the long-term memory representations relevant for auditory representations of language (cf. Mesgarani et al., 2014).^1^

The point of contention across different theoretical models built around the notion of *distinctive features* is how to best characterize their nature and their mental organization. Early theories posited that features were loosely grounded around acoustic and articulatory information that was binary in nature (Jakobson et al., 1951; Chomsky and Halle, 1968). For example, the distinction between segments [s] and [z] was simply that the former had a negative specification for the vibration of the vocal cords, coded as [-VOICE], while the latter had a positive specification of the same articulator, [+VOICE]. The same feature distinguishes English [t] and [d], despite the fact that, in English, there is often little or no vocal fold vibration associated with [d]. A more accurate representation of the English contrast, then, is with the phonemes /t^h^/ and /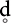/ rather than /t/ and /d/^2^. More recently, phonological theory has moved away from using binary features in favor of privative features (e.g., where [z] is specified for [VOICE] whereas [s] lacks a specification and thus lacks vocal fold vibration), arguing that the negative specification is not needed when writing phonological rules or constraints, but this difference is in principle one of notation, as any binary feature system can be recoded as a privative feature system. This abstractness of the connection between the phonetic reality and phonological features has been often repeated by phonologists, even when they use non-binary or privative features (Lombardi, 1991/1994).

Other theoretical models have explored variations on this basic representational schema, particularly a closer relationship between distinctive articulatory features in long-term (phonological) memory and their articulatory realizations (Lisker and Abramson, 1964; Iverson and Salmons, 1995; Honeybone, 2005). In these theories, some features may be tied to language-specific properties, such as exactly how a voiced/voiceless contrast is made. Laryngeal realism, for example, suggests that a language like German can be better explained when its voiced/voiceless contrast can be construed as an aspirated/unaspirated contrast (Iverson and Salmons, 1995, 1999, 2003; Honeybone, 2005).

These two kinds of theories about the connection between phonological features and their phonetic realization make divergent predictions when it comes to the laryngeal articulators. For example, many languages, like Spanish, French, Russian, English, German, Swedish, and Turkish, exhibit a two-way phonological contrast between what are traditionally described as voiced and voiceless stop consonants like /d/ and /t/. Under early, more abstract feature models, a single, binary feature, such as [+VOICE] vs. [-VOICE], would be enough to account for all these cases. However, the actual articulatory gestures that speakers of these languages use to produce these two-way distinctions are known to vary cross-linguistically. Some languages, like Spanish, French, and Russian, use primarily the timing of the onset of vocal fold vibration—voice onset time (VOT)—before the consonant release to mark the two-way distinction: they contrast pre-voiced stops with neutral or shot-lag stops. Other languages, like English and German, mark a two-way distinction primarily with aspiration, a long lag between the stop release and the onset of voicing, contrasting a plain or short-lag consonant with a long-lag one (see Lisker and Abramson, 1964). These different phonetic details can be captured by a system involving an inventory of laryngeal articulators, such as [VOICE] (which controls the vibration of the vocal cords) and [SPREAD GLOTTIS] (which controls the amount of aspiration), each of which may have positive or negative values (under a binary feature approach) or be specified or left unmarked (under a privative feature approach). In a true voicing language like Russian (Petrova et al., 2006; Ringen and Kulikov, 2012; Nicolae and Nevins, 2015), [VOICE] would be the active feature responsible for the two-way distinction, whereas in languages like English and German, this role would be accomplished by [SPREAD GLOTTIS].

Therefore, different feature models make different predictions about the underlying structure and representation of laryngeal articulatory features. Early theories predict a simple binary distinction that abstracts from significant articulatory detail in order to implement a simple two-way phonological contrast. More recent theories, on the other hand, propose that simple two-way phonological contrasts can be implemented by different combinations of a richer set of underlying articulatory features, and that these combinations can vary across languages.

In this paper, we turn to neurophysiological data, in the form of the Mismatch Negativity (MMN) paradigm, that has been argued to reveal at least some aspects of phonological structure (Phillips et al., 2000; Walter and Hacquard, 2004; Kazanina et al., 2006; Scharinger et al., 2010, 2012; Cornell et al., 2011, 2013; Law et al., 2013; Truckenbrodt et al., 2014; de Jonge and Boersma, 2015; Hestvik and Durvasula, 2016; Politzer-Ahles et al., 2016; Schluter et al., 2016) in order to test these different representational approaches. In three MMN experiments, we test English, Arabic, and Russian, three different languages that have a functional two-way voicing distinction at a phonological level, but which rely on different underlying articulatory mechanisms to implement these distinctions during speech production. If earlier feature models are correct and the long-term feature representation abstracts away from considerable phonetic detail, then we predict a stable cross-linguistic pattern in the results across languages (English, Arabic, and Russian) and across consonant types (fricatives and stops). If, on the other hand, the long-term representation of laryngeal features is more closely tied to their precise articulatory detail, we predict different cross-linguistic patterns, since these languages’ respective two-way voicing distinctions are implemented via the use of differently specified laryngeal articulators.

### Phonetics and Phonological Representations

**FIGURE 1:**
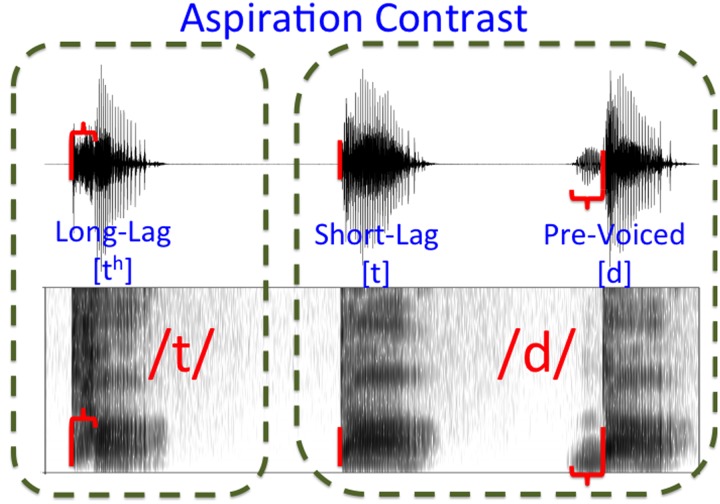
**Aspiration Contrast**. The boundary between the three major voice onset time categories separates long-lag stops from short-lag and pre-voiced stops. Release time is indicated with a vertical red bar, with aspiration or pre-voicing represented as a horizontal bracket.

Given that there are multiple ways to implement a two-way contrast, we are interested in the question of whether languages use one relatively phonetically abstract feature to do this, or if phonetically distinct contrasts are encoded in different ways. Two types of obstruent consonant commonly display a voicing contrast: stops and fricatives.^3^ Fricatives such as [f], [v], [s], and [z] are distinguished in terms of voicing by the presence or absence of vocal fold vibration^4^. Stop consonants, however, are often described in terms of a VOT continuum in which the difference between voiced and voiceless can vary depending on where the categorical boundary lies (Lisker and Abramson, 1964; Beckman et al., 2011; Beckman et al., 2013). Pre-voiced stops (with negative VOT as the voicing gesture begins before the release of the consonant) may contrast with plain or short lag VOT consonants (with the release occurring concurrently or shortly before voicing begins) or long-lag VOT consonants (with the release occurring well before voicing begins). Thus, for any given language a two-way stop contrast may have one of three articulatory-phonetic patterns: pre-voiced vs short-lag (Spanish, French, and Russian), short-lag vs. long-lag (English, German), or pre-voiced vs. long-lag (Swedish, Turkish). The difference between aspiration and pre-voicing languages is shown in **Figure 1** (aspiration) and 2 (pre-voicing). Other languages even use a three way contrast: pre-voiced vs. short-lag vs. long-lag (Thai).^5^ Nonetheless, in terms of long-term mental representations, phonologists tend to use the same features to represent the voice-voiceless contrast in stops as they do for fricatives because it is the categorical contrast that is seen as ultimately creating a coherent mental representation for the entire sound system^6^. Therefore, there are two issues at play when capturing the complexity of a two-way contrast in phonology: (1) the number of features used, and (2) the values of those features.

**FIGURE 2:**
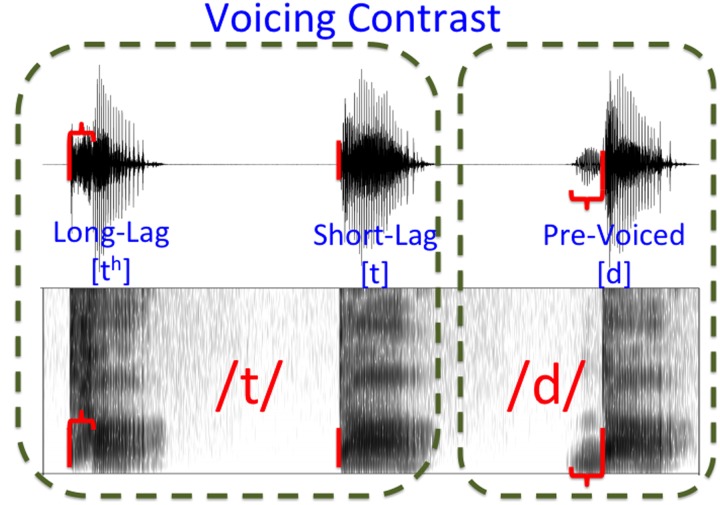
**Voicing Contrast**. The boundary between the three major voice onset time categories separates pre-voiced stops from short- and long-lag stops. Release time is indicated with a vertical red bar, with aspiration or pre-voicing represented as a horizontal bracket.

The number of features speaks to how abstract the relationship between phonetics and the mental representations are. In a one-feature system, the feature’s presence or absence in the mental representation is enough to distinguish two sounds, but not to clearly spell out the phonetic implementation. For example, in English one feature could be used to distinguish the abstract relationship between /t^h^/ and /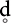/ (aspiration) and the relationship between /s/ and /z/ (vocal fold vibration). Similarly one feature could capture the difference between Russian where the distinction between /t/ and /d/ is pre-voicing rather than aspiration. If this is true, we expect that we can get the same results by testing stops and fricatives in a comparable way, and testing typologically distinct languages for the same results.

The second issue is the label of the features and the label’s relationship to articulation and acoustics. While one abstract feature could be labeled in any way, phonologists have long suspected that the physical implementation of language should be taken into account when labeling these features (see, e.g., Lombardi, 1991/1994, and references therein). Thus, the contrast in English might be labeled with a feature related to the vibration of the vocal folds—[VOICE]—or alternately with reference to the absence of these vibrations. Where the absence of vibrations may seem odd from a physiological level at first, preventing the vocal folds from vibrating during speech does require muscular effort to keep the vocal folds apart and has distinct acoustic contributions to the speech signal (Edmondson and Esling, 2006). Thus, a feature referring to the muscular effort to keep the vocal folds from vibrating—[SPREAD GLOTTIS]—could be used as the label for the same contrast. While the specific labels and machinery for these features may vary (cf. Jakobson et al., 1951; Halle, 1959, 2005; Chomsky and Halle, 1968; Avery, 1997; Avery and Idsardi, 2001; Gallagher, 2011, among numerous others), we adopt the well-known labels *voice* and *spread glottis* (cf. Lombardi, 1991/1994).

A third issue is the valuation of these labeled features. There is considerable debate among phonologists if features should be coded as binary (i.e., [+VOICE] vs. [-VOICE]) or if privative features (i.e., [VOICE] vs. [ ]) are able to encode the same two-way distinction as [+VOICE] vs. [-VOICE]. Here, we largely ignore this debate as it is somewhat orthogonal to our research question. Whether the phonological system of a language needs to refer to both the positive and negative values of a feature is at the heart of this debate, and we note that there is some recent literature suggesting a need for a reference to both labels of a binary feature, for e.g., that [-VOICE] is necessary to represent phonological processes in some languages (Wetzels and Mascaró, 2001; Bennet and Rose, unpublished). More relevant for our purposes is the notion of markedness, that one of the two options (i.e., [+VOICE] vs. [-VOICE] or [VOICE] vs. [ ]) is marked (i.e., specified with a feature) while the other is unmarked (i.e., left featurally unspecified). A marked feature is seen as phonologically active, while the unmarked option would be phonologically inert. These correlate to some extent with the neurophysiological results of Eulitz and Lahiri (2004) and we adopt their logic regarding feature specification^7^. Thus, we currently ignore the issue of what it might mean for a feature to be marked in the negative or unmarked, in favor of focusing on marked and privative feature labels. We further simplify our terminology for expository purposes and will simply refer to marked or unmarked features henceforth.

A tight correlation between phonetics and phonology has been argued in the form of laryngeal realism (Iverson and Salmons, 1995, 1999, 2003; Honeybone, 2005). Laryngeal realism states that the phonetics of a voiced-voiceless contrast indicate the feature marking responsible for the contrast. An aspirating language like German or English will mark the contrast with a feature responsible for aspiration [SPREAD GLOTTIS] while a voicing language like Spanish or Russian will mark the contrast with a [VOICE] feature. Using our terminology laid out above, this would mean that languages like English and German, on the one hand, would have phonemes traditionally described as voiceless (like /p/, /t/, and /k/) bearing a *marked* laryngeal feature [SPREAD GLOTTIS], and their traditionally described as voiced counterparts (like /b/, /d/, and /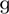/) left unmarked for their laryngeal gestures. In voicing languages like French or Russian, on the other hand, the situation would be reversed: phonemes traditionally described as voiceless (like /p/, /t/, and /k/) would be left unmarked, and the traditionally described as voiced (like /b/, /d/, and /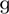/) would be marked for [VOICE].

Many recent phonetic studies (Helgason and Ringen, 2008; Beckman et al., 2011, 2013; Ringen and Kulikov, 2012; Ringen and van Dommelen, 2013; Nicolae and Nevins, 2015) find support for laryngeal realism, providing evidence, for instance, that rate of speech affects the pronunciation of the marked stop (i.e., pre-voicing or long-lag duration) but not the unmarked, short-lag stop. Indeed, in Swedish, this is taken as evidence for contrastive overspecification, as dialects of Swedish and Norwegian phonetically contrast pre-voiced with long-lag stops. The logic underlying these studies is that rate of speech should only cause changes to segments bearing the marked feature value because these are actual gestural commands; the neutral, short-lag stop is a sort of default without any particular articulatory gesture associated with it.

These articulatory results—consistent with laryngeal realism—are also consistent with data from language acquisition. Kager et al. (2007) also tested some of the predictions of laryngeal realism by analyzing speech errors in English, German, and Dutch. Assuming the phonetically grounded articulatory feature representation used by laryngeal realism, Kager et al. (2007) hypothesize that children ought to make more speech errors toward the unmarked, rather than the marked segment. Contrasting a voicing language (Dutch, which putatively marks [VOICE]) with aspirating languages (English and German, which putatively mark [SPREAD GLOTTIS]), Kager et al. (2007) find that Dutch children make more speech errors toward voiceless segments and that English and German children make more errors toward voiced ones. Kager et al. (2007) argue that a mixed analysis where the marked feature differs from language to language makes better predictions than one in which only one feature (e.g., [VOICE]) is used for all three languages.

Whether [VOICE] or [SPREAD GLOTTIS] is active in English, however, is not uncontroversial. Kohn et al. (1995) argue that evidence from aphasic disfluencies suggest that voiced consonants of English are marked rather than voiceless ones, whereas laryngeal realism would posit the opposite if [SPREAD GLOTTIS] is the marked feature responsible for the English two-way contrast, under the assumption that only a marked feature should be active in the phonology of the language. The aphasic patients in Kohn et al. (1995)’s study tended to erroneously substitute the homorganic [+VOICE] consonant when another [+VOICE] consonant occurred in the same word, indicating that [VOICE] active in the phonology, and therefore had a marked value. This was not true for their [-VOICE] or [SPREAD GLOTTIS] consonant errors (i.e., [f𝜀s] for *vest* was an uncommon error type while [gælevin] for *calendar* was significantly more common). In a similar vein, Hwang et al. (2010) find evidence that it is the voiceless segment (e.g., English /t/) that is unmarked, because it fails to produce predictions in the perception of final consonant clusters. In a conscious categorization task, the voiced-voiceless sequence (e.g., [uds]) is responded to more slowly and less accurately than codas matching in terms of laryngeal state (i.e., [uts], [udz]) or the voiceless-voiced sequence (i.e., [utz]). The slower and less accurate member of the quadruplet is theorized to be distinct as the voiced stop induces a prediction for a following voiced fricative (assumed to be marked for [VOICE]) which is violated in the [ds] sequence. Moreover, Vaux (1998) argues that, cross-linguistically, it is the voiceless fricative that is marked, except in languages like Burmese which contrast voiced /z/, voiceless /s/, and voiceless aspirated /s^h^/ fricatives. Recent neurophysiological evidence, however, has been argued to support the laryngeal realism hypothesis (Hestvik and Durvasula, 2016).

### Mismatch Negativity (MMN)

Research on electrophysiology of language has revealed the potential sensitivity of an event-related potential called the MMN to phonological structure (Dehaene-Lambertz, 1997; Phillips et al., 2000; Eulitz and Lahiri, 2004). The MMN (and its magnetoencephalography correlate, the mismatch field or MMF; Näätänen, 2001; Näätänen et al., 2007) is an early ERP component that is known to be sensitive to acoustic changes in general (Näätänen et al., 1978) but which has also been shown to be sensitive to categorical changes in speech stimuli (e.g., Dehaene-Lambertz, 1997; Näätänen and Alho, 1997). The MMN is usually evoked in an oddball paradigm, where a number of ‘standard’ sounds are played repeatedly and occasionally a ‘deviant’ or oddball sound is played (generally at a ratio of about seven standards per one deviant). The MMN is maximal at fronto-central sites (often Fz), and obtained by subtracting the average response to standards of one stimulus or category of stimuli from the average response to the same stimulus or category of stimuli presented as a deviant. The elicitation of an MMN indicates that the processing system has detected a change in a stream of stimuli. This change-detection property has been exploited in studies interested in investigating whether the MMN can be used to detect not only changes at an acoustic or phonetic category level, but also at a phonological level. For example, Kazanina et al. (2006) found that a robust MMN response to the voicing contrast between [d] and [t] can be observed in Russian speakers, for whom the contrast is phonemic, but no such contrast can be observed in Korean speakers, for whom [d] and [t] are allophones of the same underlying phonemic category. Similarly, Truckenbrodt et al. (2014) tested German nonce words in the context of word-final devoicing in a reverse oddball paradigm. In the crucial comparison where the deviant and standard could be plausibly related via word-final devoicing (standard /vuzǝ/ with deviant [vus]) there was no MMN detected for the fricative as the two fricatives were apparently categorized as the same segment given the context (other contexts, including standard /vus/ with deviant [vuzǝ] did show a MMN for the fricatives). While final devoicing may be linked to a morphophonological alternation, the lack of an MMN in final devoicing context does suggest that in some context either an asymmetric MMN or the MMN itself will not be found for voiced and voiceless speech sounds. Thus, we expect the MMN will show effects of categorical differences where warranted, and fail to show differences when the sounds are not distinct categories, even for voicing differences.

In addition to a basic sensitivity to phonological information, the MMN has been shown to reflect, in an interesting fashion, the *markedness* status of phonological features in the form of asymmetrical effects (Eulitz and Lahiri, 2004, et sqq.). Eulitz and Lahiri (2004) argue that asymmetries in the strength of the MMN arise when marked sounds and unmarked sounds are contrasted in a reverse oddball paradigm. When a marked sound is the deviant and an unmarked sound the standard, the MMN is smaller than when the unmarked sound is the deviant and the marked sound the standard. Eulitz and Lahiri (2004) argue this is related to the phonological representation of the sounds, where the marked deviant is not inconsistent with the unmarked standard, but an unmarked deviant has a phonetic representation which clashes with the marked stored representation of the standard, amplifying the strength of the MMN (see Alternatives Accounts and Politzer-Ahles et al., 2016, for a review of other factors that can cause MMN asymmetries that may not be tied to the markedness of distinctive features). This mechanism is referred to as *underspecification* in the phonological literature (Archangeli, 1984, 1988; Lahiri and Reetz, 2002, 2010; Eulitz and Lahiri, 2004, among others). Applying Eulitz and Lahiri’s (2004) logic to voicing and the feature marking hypothesis laid out by laryngeal realism, one would expect to observe, in an aspirating language like English, an asymmetry based on an aspiration or [SPREAD GLOTTIS] feature, as voicing in English is taken to be only a phonetic phenomenon. Indeed, this was recently tested with English stop consonants, where Hestvik and Durvasula (2016) find a larger MMN for the unmarked voiced deviant /d/ than the voiceless one (/t/). By the same token, in a voicing language, the prediction about the MMN asymmetry is the reverse: a larger MMN for the unmarked voiceless deviant (/t/) compared to the marked voiced deviant (/d/), as the voiced segment is marked for [VOICE] and the voiceless one left unmarked. However, although Hestvik and Durvasula’s MMN results are consistent with the predictions of laryngeal realism for a specific language (English), there is no current cross-linguistic evidence from MMN for laryngeal realism: this is the kind of evidence that we seek to adjudicate in this paper.

**FIGURE 3:**
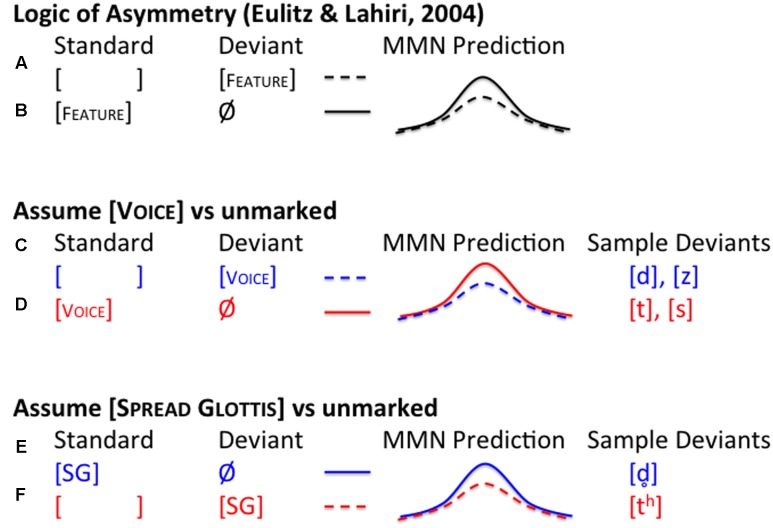
**Predictions**. When two sounds differ in markedness, a marked standard’s feature is compatible with the unmarked deviant’s lack of a feature **(a)** but the reverse is not true. An unmarked deviant **(b)** has some phonetic surface marking, which conflicts with the standard’s phonologically marked feature. This conflict causes a larger MMN. When [VOICE] is assumed to be the marked feature **(c,d)** we expect to see a different asymmetry than when [SPREAD GLOTTIS] is marked **(e,f)**. This logic can be used to determine which feature is active in a fricative contrast (e.g., [s] vs. [z]) as well as different stop contrasts (e.g., [t] vs. [d] or [t^h^] vs. [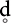]).

Here we build on the previous MMN findings to test the two different kinds of models of laryngeal feature specifications in long-term memory. Traditional single-feature models would predict that a single feature, such as [VOICE], is the relevant one responsible for the contrast in both stops and fricatives. The *laryngeal realist* theory, on the other hand, predicts a different pattern of results (see **Figure 3**). By applying the same logic of underspecification to glottalic states, in an aspirating language like English we should observe an MMN asymmetry based on an aspiration or [SPREAD GLOTTIS] feature and a voicing feature if voicing in English stops is the result of only a surface phonetic specification. The feature responsible for voicing in English fricatives, however, may differ from the [SPREAD GLOTTIS] feature used for stops. Furthermore, speakers of a voicing language should show a different pattern based on the phonetic implementation of the stop contrast: speakers of a voicing language that marks a stop contrast with pre-voicing should use a [VOICE] feature to mark the difference, not [SPREAD GLOTTIS].

### Alternatives Accounts

While we assume the underspecification mechanism of Lahiri and Reetz (2002, 2010) and Eulitz and Lahiri (2004), there are other factors which may play a role in the MMN and MMN asymmetries for both language and non-language studies. The presence or absence of an additional physical change in non-linguistic auditory or visual stimulus (relative to the standard) has been shown to produce asymmetric MMN effects (Winkler and Näätänen, 1993; Nordby et al., 1994; Sabri and Campbell, 2000; Timm et al., 2011; Bendixen et al., 2014; Czigler et al., 2014). As the N1 and MMN are temporally close to one another, differences in N1 refractoriness may modulate the responses to stimuli differentially (see May and Tiitinen, 2010, for a review). The MMN may also be influenced by differences in prototypicality (Ikeda et al., 2002) or by general perceptual biases (Polka and Bohn, 2011).

Moreover, there are some accounts which explicitly reject the proposal that underspecification can lead to MMN asymmetries to begin with. Bonte et al. (2005), for example, suggest that purportedly underspecification effects in the MMN may be due instead to uncontrolled differences in phonotactic probabilities. Tavabi et al. (2009) similarly proposed that other variables like frequency and context, rather than underspecification, may drive MMN asymmetries. Gow (2001, 2002, 2003) and Gaskell (2003) further suggest that the notion of underspecification is unnecessary for explaining alternations such as place assimilation, and Mitterer (2011) finds no evidence for underspecified representations in an eye-tracking study.

While we cannot refute all the possible objections to the linking of underspecification and asymmetric MMNs, here, we note that we specifically focus on ERPs for fricatives which are presented in isolation (excepting the stops [te] and [de] in Experiment 3) exactly to avoid many of the proposed top-down confounds above. Furthermore, we test these predictions in English, Arabic, and Russian which are typologically different in their patterns of voiced and voiceless segments, and therefore are not necessarily acoustically similar.

### Hypothesis and Predictions

Our aim is to test how close the coupling is between the phonetic implementation and long term mental representation of distinctive features, assuming the proposed link by Lahiri and Reetz (2002, 2010) and others between underspecification of phonological units and the elicitation of MMN asymmetries. We do this with three experiments. In Experiment 1, we use English fricatives to test if the feature marking of these segments is the same as the one in stops (as revealed by the results of Hestvik and Durvasula, 2016)^8^. We test this using an oddball paradigm with the English segments [f] (voiceless) and [v] (voiced) and compare the results to those of Hestvik and Durvasula (2016) for the English stops [t^h^] and [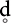]. If the same MMN pattern observed by Hestvik and Durvasula (2016) for [t^h^] and [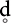] emerges for the fricatives [f] and [v] (i.e., if [v] deviants in the context of [f] standards elicit a greater MMN than [f] deviants in the context of [v] standards), we can conclude that English is likely to mark both voicing contrasts in the same way (supporting a one-feature theory, but less clearly compatible with theories like laryngeal realism, that posit a closer connection between phonetic and phonological representations). Alternatively, if the results for the fricatives [f] and [v] go in the opposite direction from the stop results observed by Hestvik and Durvasula (2016), we can conclude that the two-way voicing distinction in English stops is implemented differently, at a featural level, from the two-way voicing distinction in English fricatives, which may indicate the need to invoke two different features to account for the results; for example, [SPREAD GLOTTIS] is marked for stops, but [VOICE] is marked for fricatives.

In Experiment 2, we test whether the fricatives of English (an aspirating language) are marked in the same way as the fricatives of Arabic (a purportedly voicing language). We test both English and Arabic tokens at two places of articulation (dental [s] and [z] and interdental [θ] and [ð]) for both English and Arabic speakers. If Arabic is truly a voicing language and marks [VOICE] rather than [SPREAD GLOTTIS], we should find an interaction such that the MMN asymmetries are opposite in English and Arabic speakers, indicating that one’s native language influences the features used to represent the contrast. If we find the same pattern of asymmetries for Arabic and English speakers, we would suspect that typologically different languages may still use one set of features, not necessarily driven by the precise articulatory phonetic details of the language.

Finally, we examine the marking of both fricatives and stops in Russian (an uncontroversial voicing language, using dental fricatives /s/ and /z/, a mixed set of voiced (/v/, /z/, /ʐ/) and voiceless (/f/, /s/, /ȿ/) fricatives, and stops (/te/, /de/) to consolidate the results for fricatives and compare them directly to stop consonants. If the pattern of results for Russian fricatives is the same as English fricatives, we find support for a theory according to which fricatives are marked in the same way for these typologically distinct languages, regardless of how these languages implement the laryngeal marking of their stop consonants. Comparison to the stops will crucially suggest whether laryngeal realism is supported or not for stop consonants, as this theory posits that a voicing language would mark its voiced stops, rather than their unvoiced ones. Thus, if the feature marking hypothesis of laryngeal realism is correct, one would expect that the results observed for Russian stops will be the exact opposite pattern from the results of Hestvik and Durvasula (2016). If, on the other hand, the same pattern of MMN asymmetries is observed across English and Russian stop consonants, then a single feature may be responsible for the cross-linguistic results, in which case the value of that feature, which Hestvik and Durvasula (2016) identified as [SPREAD GLOTTIS], and the support that it lent to laryngeal realism would have been entirely coincidental, due to the fact that English was the only language investigated by Hestvik and Durvasula (2016).

## Experiment 1: English [f] vs. [v]

### Methods

#### Participants

Twenty-nine native English-speaking participants took part in the study, for which the goal was to have data from 24 subjects. Two were eliminated because of technical errors and three were eliminated because they had fewer than 30 artifact-free deviant trials in one of the blocks, leaving 24 subjects in the analysis (10 males, 14 females, mean age = 20.9, *SD* = 3.7; age data from one participant is not available). The participants were recruited from the New York University Abu Dhabi community. All participants reported normal hearing and cognitive function. Though all participants reported English dominance, seven reported some degree of bilingualism (Hindi, Urdu, Mandarin, German, Japanese, and French). All methods for the study were approved by the Institutional Review Board of New York University Abu Dhabi. Participants were compensated for their time.

#### Stimuli

Stimuli consisted of short tokens of English fricatives [f] and [v] pronounced by one female native English speaker in a sound-attenuated room. Stimuli were recorded using an Electro-Voice RE20 cardioid microphone, and digitized at 22050 Hz with a Marantz Portable Solid State Recorder (PMD 671). There were no surrounding vowels for any tokens. The use of naturally produced fricatives in isolation mirrors previous studies using vowels (Cornell et al., 2011; de Jonge and Boersma, 2015) and also eliminates any possible effects of coarticulation or phonotactic knowledge (Bonte et al., 2005), or cross-splicing (Steinberg et al., 2012), and has been successfully used in previous experiments (Schluter et al., 2016). For each type, six distinct tokens were selected by a trained phonetician. Tokens were modified in Praat (Boersma and Weenink, 2013) to a duration of about 250 ms by removing material from the middle of the token at zero-crossings, and then normalized for amplitude to 70 dB_SPL_ (RMS). Tokens were not ramped; the natural onset and offset were retained. See Supplementary Materials for audio stimuli used in this experiment.

#### Experimental Procedure

The electroencephalogram (EEG) was obtained during an oddball paradigm in one 2-hour session, concurrent with five similar experiments (not reported here). The experiment consisted of two blocks. One block contained 680 standard [v] tokens with 120 deviant [f] tokens, with an additional 20 standards at the beginning of the block. Tokens were jittered with a 400–600 ms ISI and pseudorandomized such that 2–10 standards occurred before each deviant. This allowed us to run a large number of experiments (not reported here) on the same participants on a reasonable amount of time. A second block was run with [f] as the standard and [v] as the deviant and otherwise identical. The blocks were presented to subjects in random order. Subjects watched a muted film with English subtitles during the experiment and were offered a break after each block.

#### EEG Acquisition and Preprocessing

EEG was continuously recorded from 34 active Ag/AgCl electrode positions (actiCAP, Brain Products) using a BrainAmp DC amplifier (Brain Products). The sampling rate was 1000 Hz, and data were filtered online from 0.1 to 1000 Hz. FCz served as the online reference and AFz as the ground. Interelectrode impedances were kept below 25 kΩ. Subjects were asked to sit still and avoid excessive eye movements.

Offline data was re-referenced to the average of both mastoids and band-passed filtered at 0.5–30 Hz for each participant. The data were segmented into 701 ms epochs (-200 to 500 ms). The initial set of 20 standards, the first deviant in each block, and the first standard after each deviant were excluded from further analysis. Epochs were baseline-corrected using a 100 ms pre-stimulus interval. Epochs with voltages exceeding ±75 μV on any channel were removed from analysis. For each participant at least 30 deviant trials per condition were retained. The MMN was calculated by subtracting the average ERP response to each standard from the average ERP response to the same stimulus type as a deviant in the other block: e.g., standard [f] from one block was subtracted from deviant [f] from the other.

Statistical analysis of MMN amplitude was conducted via spatiotemporal cluster-based permutation tests (Maris and Oostenveld, 2007) over the 100 to 300 ms post-stimulus-onset time window (a broad window in which the MMN is expected to appear). This method checks for clusters of spatially and temporally adjacent data point clusters that meet an arbitrary threshold of significance (*p* = 0.05) and then evaluates the significance of these clusters using a non-parametric permutation statistic. While the MMN has a well-known time-course and topography (Näätänen and Alho, 1997; Näätänen, 2001; Näätänen et al., 2007), this statistical analysis reduces (but does not eliminate) researcher degrees of freedom in the choice of analysis window, as it allows for testing main effects over a broad temporal and spatial window and makes use of all 31 channels used in the analysis rather than only one.

### Results

**FIGURE 4:**
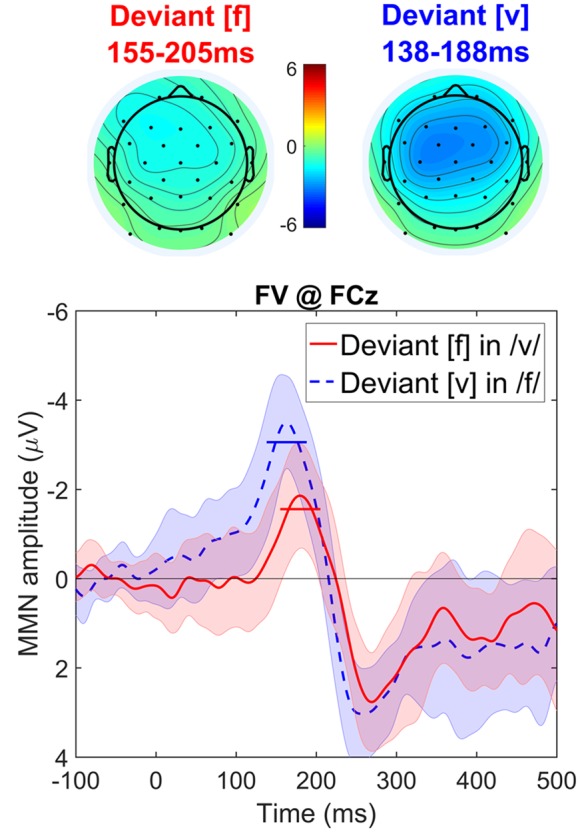
**Topographic maps and difference waves (at Fz) for deviant [f] (red) and deviant [v] (blue)**. Ribbons indicate a difference-adjusted 95% Cousineau-Morey within-subjects interval (which can be interpreted as follows: at a given time point, if neither condition’s difference-adjusted interval contains the other condition’s mean, then the difference between conditions is likely to be significant at the 95% alpha level, without correction for multiple comparisons). Horizontal lines on the difference waves indicate the average amplitude for the 51 ms window centered on the MMN peak.

Visual inspection of the data (see **Figure 4**) suggests the two conditions are distinct, and that deviant [v] evokes a greater MMN than deviant [f]. This asymmetry is consistent with the results of Hestvik and Durvasula (2016) as our voiced deviant fricative [v] patterns with their voiced stop and vowel stimulus [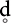
æ] and our voiceless [f] with their [t^h^æ].

The cluster-based permutation test revealed significant differences between the MMNs elicited by voiced and voiceless deviants. Voiced deviants elicited more negative MMNs than voiceless deviants (*p* < 0.001) based on a cluster of samples from 100 to 185 ms and including 25 channels: Fp1, F3, Fz, F4, FC5, FC1, FC2, FC6, T7, C3, Cz, C4, CP5, CP1, CP2, CP6, P7, P3, Pz, P4, P8, O1, Oz, O2, and FCz. Voiced deviants also elicited more positive later effects than voiceless deviants (*p* = 0.009), based on a cluster from 212 to 300 ms and including 20 channels: Fp1, Fp2, F7, F3, Fz, F4, F8, FC5, FC1, FC2, FC6, C3, Cz, C4, T8, CP1, CP2, CP6, P4, and FCz (i.e., the P3 wave following the MMN).

### Discussion

The results here show an asymmetry between the MMN magnitude observed for the voiced [v] vs. voiceless [f], and are in line with a long-term encoding system in which the voiced segment is unmarked and the voiceless segment is marked, as indicated by the predicted asymmetric MMN patterns (cf. Eulitz and Lahiri, 2004; Scharinger et al., 2010, 2012; Cornell et al., 2011, 2013; de Jonge and Boersma, 2015; Schluter et al., 2016). Furthermore, the more negative peak for the voiceless deviant suggests that it is the voiceless sound which is marked for English fricatives, just as Hestvik and Durvasula (2016) found for English stops. These results may suggest that one single feature accounts for both English stops and fricatives and, following the feature marking hypothesis of laryngeal realism, that feature should be [SPREAD GLOTTIS]. Alternatively, contrary to the feature marking hypothesis of laryngeal realism, it may be the case that the feature specification for English voicing may coincidentally be a universal marking. Cross-linguistic evidence is required to determine if other languages use a [VOICE] feature in lieu of [SPREAD GLOTTIS]. Such evidence would be found if the voiced deviant were to show a *smaller* MMN than the voiceless one in a language hypothesized to use [VOICE] rather than [SPREAD GLOTTIS] to distinguish a two-way voicing contrast.

In the next experiment, we seek to replicate these English results with other places of articulation and compare the asymmetry for English (an aspirating language) with Arabic (purportedly a voicing language). Given how the functional two-way voicing contrast in these two languages is phonetically realized by different articulatory means (unmarked [VOICE] and marked [SPREAD GLOTTIS] in English, and marked [VOICE] and unmarked [SPREAD GLOTTIS] in Arabic), a theory of distinctive features that posits a strong connection between articulatory detail and the long term distinctive feature representation would predict the opposite patterns of MMN asymmetries in these two languages. If, however, the functional two-way contrast abstracts away from this level of phonetic detail, the MMN asymmetric patterns are predicted to be similar across these two languages. We test these competing predictions with fricative sounds possessing two other places of articulation: dental ([s] and [z]) and interdental ([θ] and [ð]). These two places of articulation occur in both Standard English and Emirati Arabic and allow us to see whether the predicted asymmetries are robust across segments varying in place of articulation.

## Experiment 2: English And Arabic Voiced vs. Voiceless Fricatives [s], [z], [θ], and [ð]

### Methods

#### Participants

We sought to test 24 participants in each language group. To that end, 27 native English-speaking participants took part in the study. The three participants with the lowest number of artifact-free deviant trials in any given block (less than 32) were eliminated, leaving 24 subjects in the analysis (13 males, 11 females, demographic information on age is unavailable for 1 participant: mean age = 20.6, *SD* = 3.4). The English-speaking participants were recruited from the NYUAD community and from among primary and secondary teachers in Abu Dhabi. Thirty-three native Arabic-speaking participants participated in the study. Three were eliminated for technical problems during data acquisition, and 6 because they were speakers of Arabic dialects other than Emirati, or because they were early English-Emirati Arabic bilinguals, leaving 24 participants whose data were analyzed in the study (24 females^9^, mean age = 22, *SD* = 1.8). The Arabic-speaking participants were all recruited at the United Arab Emirates University and reported that their parents pronounced the letters [scale=.50]img002 and [scale=.50]img003 in the classical way (i.e., as [θ] and [ð]; Emirati Arabic speakers tend to pronounce them as fricatives but the pronunciation is more varied among other dialects of Arabic with some dialects using stops ([t] and [d]) and others dental fricatives ([s] and [z]). All participants reported normal hearing and cognitive function. Ten of the English-speakers reported some degree of bilingualism (American Sign Language, Arabic, French, Korean, Japanese, Mandarin, and Spanish). All Arabic speakers were bilingual in English (the language of instruction at the United Arab Emirates University) but reported late bilingualism (they learned English in school, rather than at home). The study protocol was approved by the Institutional Review Board of New York University Abu Dhabi and the Ethics Committee at the United Arab Emirates University, and participants were compensated.

#### Stimuli

Stimuli consisted of short tokens of English fricatives (without following vowels) [s], [z], [θ], and [ð] pronounced by one female native English speaker in a sound attenuated room and Arabic fricatives [s], [z], [θ], and [ð] pronounced by one female Emirati Arabic native speaker in a sound attenuated room. Tokens were shortened to 250 ms by removing medial material at zero-crossings and normalized for intensity to 70 dB_SPL_. For each type, six distinct tokens were selected by a trained phonetician. See Supplementary Materials for audio stimuli used in this experiment.

#### Experimental Procedure

The experimental procedure was identical to that of Experiment 1, except the Arabic-speaking participants watched a film with Arabic subtitles while English speakers watched a film with English subtitles.

#### EEG Acquisition and Preprocessing

The acquisition and preprocessing of the EEG were the same as Experiment 1, except that the online filter was set to 0.01–250 Hz, and two different EEG systems housed at different locations (one at NYUAD and the other at UAEU) were used for data collection. They were otherwise the same models produced by the same manufacturer (BrainAmp DC amplifier, Brain Products), using the same models of active electrode caps (actiCAP with 34 active Ag/AgCl electrodes, Brain Products).

#### Data Analysis

The results were analyzed using cluster-based permutation tests, as in Experiment 1. Even though the design of the experiment could be analyzed by means of a the 2 × 2 × 2 × 2 repeated measures mixed ANOVA (SPEAKERLANGUAGE: English, Arabic; TOKENLANGUAGE: Native, Non-native; PLACEOFARTICULATION: Interdental, Dental; LARYNGEALSTATE: Voice, Voiceless), presenting the results of such a high-order model is notoriously challenging. Here, for ease of exposition we opt instead for four planned pairwise comparisons, but the reader interested in the full 2 × 2 × 2 × 2 repeated measures ANOVA is referred to the Supplementary Materials.

### Results

**FIGURE 5:**
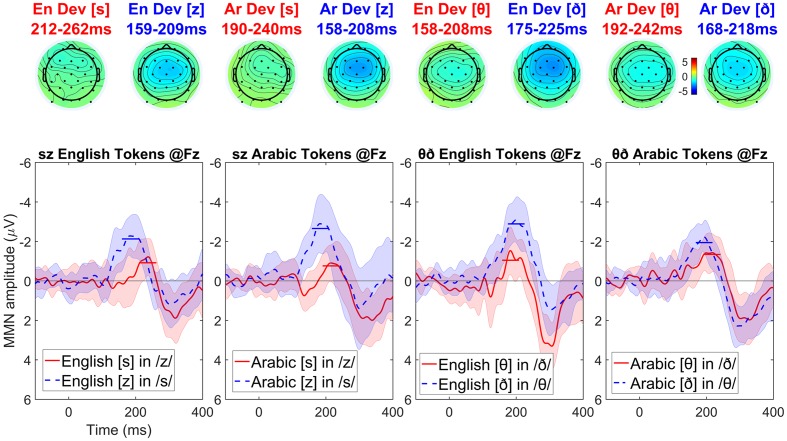
**Topographic maps and difference waves (at Fz) for voiceless deviants (red) and voiced deviants (blue) for English speakers**. Ribbons indicate a difference-adjusted 95% Cousineau-Morey within-subjects interval (which can be interpreted as follows: at a given time point, if neither condition’s difference-adjusted interval contains the other condition’s mean, then the difference between conditions is likely to be significant at the 95% alpha level, without correction for multiple comparisons). Horizontal lines on the difference waves indicate the average negativity for the 51 ms window centered on the MMN peak.

**FIGURE 6:**
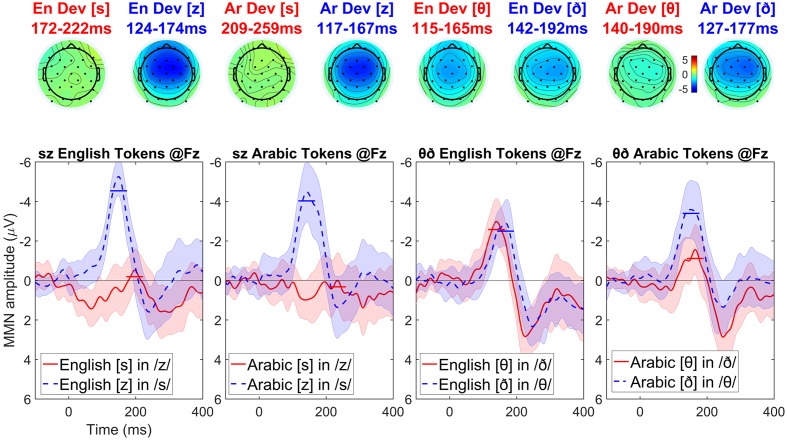
**Topographic maps and difference waves (at Fz) for voiceless deviants (red) and voiced deviants (blue) for Arabic speakers**. Ribbons indicate a difference-adjusted 95% Cousineau-Morey within-subjects interval (which can be interpreted as follows: at a given time point, if neither condition’s difference-adjusted interval contains the other condition’s mean, then the difference between conditions is likely to be significant at the 95% alpha level, without correction for multiple comparisons). Horizontal lines on the difference waves indicate the average negativity for the 51 ms window centered on the MMN peak.

Visual inspection of the English-language participants’ data (see **Figure 5**) suggests the same asymmetric MMN pattern is obtained across the pairs of stimuli: voiced deviants elicit a stronger MMN than the voiceless ones (although the magnitude of this asymmetry is smaller in the Arabic interdentals compared to the other stimuli). The symmetric MMN amplitudes for the Arabic interdentals may result from these being bad exemplars of interdentals for English speakers. Visual inspection of the Arabic-language participants’ data (see **Figure 6**) shows a very similar pattern as the one observed in English: voiced deviants elicit a stronger MMN than the voiceless ones. The exception is again an interdental stimulus pair, but this time it is the English set that shows symmetric MMN effects. The symmetric MMN amplitudes for the English interdentals may result from these being bad exemplars of interdentals for Arabic speakers, and would be the mirror image of the pattern found for the interdental stimuli in the English-language group.

For English-speaking listeners, the MMN elicited by voiced deviants in the context of voiceless (putatively marked) standards is expected to be more negative than the MMN elicited by voiceless deviants in the context of voiced (putatively unmarked) standards. Thus, the difference wave of the voiced MMN minus the voiceless MMN is expected to be *negative*. For Arabic-speaking listeners, under the laryngeal realism hypothesis, the asymmetry is expected to be in the opposite direction: the difference wave of the voiced MMN minus the voiceless MMN is expected to be positive. [Alternatively, other non-phonological perceptual factors may exert similar influences on both English-speaking and Arabic-speaking listeners (see e.g., Politzer-Ahles et al., 2016), which may cause this difference wave to be negative for Arabic-speaking listeners as well, but at least it should be *less* negative than that for English-speaking listeners]. Therefore, if the feature marking hypothesis of laryngeal realism are correct, the difference of the MMN waves for English-speaking listeners minus the difference of the MMN waves for Arabic-speaking listeners must be *negative-going*. The cluster analysis, therefore, focused on testing for such negative-going differences, i.e., comparisons in which the difference of MMNs was more negative for English-speaking than Arabic-speaking listeners.

To do this we conducted four between-group comparisons, comparing the difference of MMNs in English-speaking and Arabic-speaking listeners for four conditions: English dental tokens, English interdental tokens, Arabic dental tokens, and Arabic interdental tokens. For simplicity’s sake we conducted this as four pairwise comparisons rather than as a factorial analysis. Running four uncorrected pairwise comparisons is anticonservative, increasing the likelihood of finding the difference that is predicted by laryngeal realism; in other words, this analysis stacks the deck in favor of laryngeal realism, so if the result fails to support laryngeal realism this could not be due to using anticonservative statistics.

**FIGURE 7:**
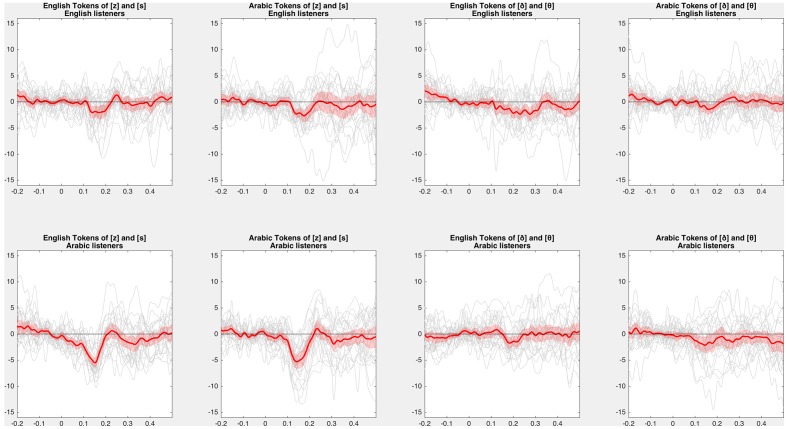
**Difference of difference waves**. Average (red) and individual (gray) difference of difference waves for each contrast. Difference of differences (voiced deviant in voiceless standard minus voiceless deviant in voiced standard) in each condition show a negative deflection in the MMN window. Red shaded ribbons represent 95% confidence intervals (i.e., the standard error of the grand average times the critical *t*-value with 23 degrees of freedom) of the MMN asymmetry.

The cluster-based permutation test used the same settings as in Experiment 1, except that the clustering statistic was an independent *t*-test rather than a dependent *t*-test. No significant negative-going differences were found between the English-speaking and Arabic-speaking listeners in English dentals (cluster *p* = 0.451), Arabic dentals (no negative clusters), or Arabic interdentals (no negative clusters). A marginal negative-going difference was found for English interdentals (*p* = 0.065, based on a 265–300 ms cluster), where Arabic-speaking listeners had a less negative asymmetry than English-speaking listeners; the pattern of this finding (a difference at the very end of the analysis window, and not in the middle) suggests that the MMN asymmetry for English speakers was longer-lasting (beginning earlier and ending later) rather than higher in amplitude *per se* (see also **Figure 7**).^10^ In an analysis with dependent *t*-tests (testing whether the asymmetry was significant within a given language), in no condition was the Arabic-speaking listeners’ asymmetry positive (with a smaller MMN for voiced than for voiceless deviants, as predicted by laryngeal realism): the asymmetry was non-significant at *p* = 0.304 for English dentals, and no positive clusters at all were found for the other conditions.

In most cases, the asymmetry for Arabic-speaking listeners was *more* negative than for English-speaking listeners, opposite what laryngeal realism predicts. Positive-going differences between English-speaking and Arabic-speaking listeners’ asymmetries (indicating a larger asymmetry for Arabic-speaking listeners) were observed for English dentals (*p* < 0.001), Arabic dentals (*p* = 0.002), and Arabic interdentals (*p* = 0.038), although not for English interdentals (no positive clusters). This is visible in **Figure 7**, particularly for the dentals, which elicit much larger negative asymmetries for Arabic than for English.

### Discussion

In Experiment 2 we clearly observe the same MMN asymmetry pattern as reported in Experiment 1 and by Hestvik and Durvasula (2016). Thus, we find no evidence from fricatives that typologically different languages (which display pre-voicing or long-lag VOT in stop consonants) mark their fricatives differently in terms of their laryngeal features. Arabic listeners did not at all show an asymmetry in the opposite direction as English speakers. Even if one allows a modified prediction for laryngeal realism (taking into account the possibility that both language groups’ MMN asymmetries may be affected by identical perceptual factors even while they’re affected by opposite phonological factors), the prediction also finds only extremely weak support: Arabic speakers’ MMN asymmetries were not even attenuated relative to English speakers’ (except marginally so, in one out of four uncorrected comparisons in an anticonservative analysis), whereas in three out of four conditions their MMN asymmetries were unexpectedly *enhanced* relative to English speakers’.

These results support a single-feature theory for fricatives because the MMN asymmetry is the same direction for each native language contrast. In addition, accepting the feature marking hypothesis of laryngeal realism and the link between MMN asymmetries and feature marking proposed by Eulitz and Lahiri (2004) and others (e.g., Scharinger et al., 2010, 2012; Cornell et al., 2011, 2013; de Jonge and Boersma, 2015; Schluter et al., 2016), these results further suggest that the marked segments for both English and Arabic are the voiceless fricatives because the voiced deviants produce a larger MMN than the voiceless deviant do. Therefore, it seems that it is a feature like [SPREAD GLOTTIS], or another one that marks the voiceless rather than the voiced phonemes, that is responsible for the asymmetries in both English and Arabic.

Despite the seemingly clear-cut pattern of voicing asymmetries here, there are some language-internal reasons that suggest we might expect Arabic to have an active [SPREAD GLOTTIS] feature alongside an active [VOICE] feature. While one hallmark of Arabic-accented English is strong pre-voicing in English voiced stops, Emirati Arabic has a three-way coronal stop contrast that involve pharyngealization, which may involve a [SPREAD GLOTTIS] marking: /d/, /t/, and /t^ʕ^/^11^. For most Arabic speakers, the /d/ is strongly pre-voiced while the /t/ is lightly aspirated. If /t^ʕ^/ is a short-lag segment (suggested by Kulikov, 2016), aspiration may be a primary or secondary cue for /t/, unlike /t^ʕ^/. If the language has both [VOICE] and [SPREAD GLOTTIS] features active, one might argue that one feature must be encoded more strongly than the other or than a third unmarked option. Multiple active features encoded with different strengths might explain the unexpected asymmetry (though it is not entirely clear how a binary or privative theory of phonological features would have an intermediate level of activation), but it is not clear what to expect if both features are active in the language^12^.

In order to ascertain whether our results in Experiment 2 were potentially clouded by uncertainties about the typological status of Arabic laryngeal features under a multiple laryngeal articulator theory, we turn to Russian. Russian is uncontroversially a voicing language, which contrasts pre-voiced and voiceless unaspirated stops. If any language were to use an active [VOICE] feature for its stops, we expect it to be Russian (even if its fricatives may be specified as [SPREAD GLOTTIS]). Furthermore, we use a larger number and a wider variety of tokens to assess whether we are truly capturing a phonological effect, as more variation in the input seems to drive participants to access more abstract representations (cf. Phillips et al., 2000; Hestvik and Durvasula, 2016; Politzer-Ahles et al., 2016).

## Experiment 3: Russian Voiced and Voiceless Fricatives and Stops

### Methods

#### Participants

Like in the first two experiments, we sought to test 24 participants. In order to achieve this sample size, 27 native Russian speakers were recruited from the NYUAD community. Two were eliminated from the study for having fewer than 30 artifact-free deviant trials per contrast, and one for withdrawing from the study before its completion, leaving 24 participants whose data were analyzed in the study (17 female, 7 male, average age = 21.2, *SD* = 4.5). All Russian speakers were bilingual in English (the language of instruction at the NYUAD) but reported late bilingualism (they learned English in school, rather than at home; seven reported Uzbek, Belarusian, Kazakh, or Romanian being spoken at home as well, each appears to be a voicing language based on the participant’s pronunciation of several stop-initial words in their second native language). All methods for the study were approved by the Institutional Review Board of New York University Abu Dhabi, and participants were compensated.

#### Stimuli

Stimuli were constructed in the same way as Experiments 1 and 2, except one native speaker of Russian produced tokens of /f/, /v/, /s/, /z/, /ȿ/, /ʐ/, /x/, /te/, and /de/. Once again, there was no vowel for the fricatives. Stimuli were not normalized for intensity, but stimuli with voicing (including the vowel of [te] and [de] were normalized for flat pitch (about 110 Hz)). Fricatives had a 50 ms onset ramping. To ensure that enough variability was included to tap into abstract representations, 10 tokens of each type were selected at random for each type from the pool of viable recorded tokens. See Supplementary Materials for audio stimuli used in this experiment.

#### Experimental Procedure

The procedure is identical to Experiment 1, except that the participants watched a film with Russian subtitles, and blocks consisted of 142 deviants and 848 standards since there were only seven blocks. The blocks include two dental fricative comparisons ([s] vs. [z]), two mixed fricatives ({[f], [s], and [ȿ]} vs. {[v], [z], and [ʐ]})^13^, two stops with vowels ([te] and [de]), and one control block of mixed fricatives ([f], [v], [s], [z], [ȿ], [ʐ], [x]) containing about 142 instances of each sound.

#### EEG Acquisition and Preprocessing

Acquisition and processing methods are identical to Experiment 1, except the analysis lent itself to an ANOVA contrasting the factors DEVIANTLARYNGEALSTATE (Voice, Voiceless) and CONTRAST (Dental, Mixed Fricatives, Stops) for a 2 × 3 design.

### Results

**FIGURE 8:**
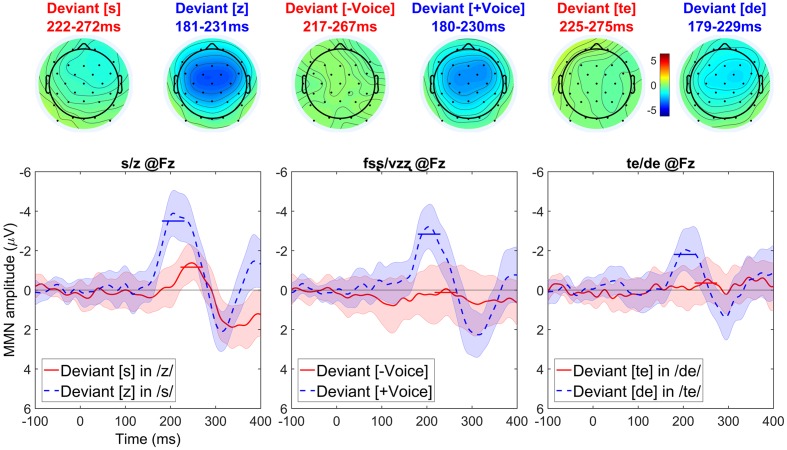
**Topographic maps and difference waves (at Fz) for voiceless deviants (red) and voiced deviants (blue)**. Ribbons indicate a difference-adjusted 95% Cousineau-Morey within-subjects interval (which can be interpreted as follows: at a given time point, if neither condition’s difference-adjusted interval contains the other condition’s mean, then the difference between conditions is likely to be significant at the 95% alpha level, without correction for multiple comparisons). Horizontal lines on the difference waves indicate the average negativity for the 51 ms window centered on the peak. Three contrasts—[s] vs. [z] (left), [fsȿ] vs. [vzʐ] (middle), and [te] vs. [de] (right)—all show the same asymmetrical pattern.

A visual inspection of the data (see **Figure 8**) suggests that asymmetries in the same direction are found regardless of segment type.

The CONTRAST × DEVIANTLARYNGEALSTATE interaction was not significant (*p* = 0.127). There was a significant main effect of CONTRAST (*p* < 0.001) but this effect is of no theoretical interest to the research question, as it is not informative about asymmetries. There were significant main effects of DEVIANTLARYNGEALSTATE. Most importantly, there was a significant negativity (*p* < 0.001), due to a cluster of samples in which voiced deviants elicited more negative MMNs than voiceless deviants; this cluster lasted from 146 to 255 ms and included 30 channels: Fp1, Fp2, F7, F3, Fz, F4, F8, FC5, FC1, FC2, FC6, T7, C3, Cz, C4, T8, CP5, CP1, CP2, CP6, P7, P3, Pz, P4, P8, PO9, O1, Oz, O2, and FCz. This main effect also showed a marginal positivity (*p* = 0.072) corresponding to the asymmetrical P3 effect that followed the asymmetrical MMN effects; this positivity was due to a cluster of samples lasting from 279 to 300 ms (the effect clearly extends beyond this time window, as shown in the plots, but the analysis window selected *a priori* was the 100-300 ms window predicted to include the MMN) and including 19 channels: F3, Fz, F4, F8, FC5, FC1, FC2, FC6, C3, Cz, C4, T8, CP1, CP2, CP6, Pz, P4, P8, and FCz.

### Discussion

Russian showed asymmetries in the same direction as English and Arabic (more negative MMNs for voiced compared to voiceless deviants) in all three contrasts tested. The uniformity of these asymmetric MMN patterns across the languages, particularly between Russian, which is well known to be a voicing language, and English, which is well known to be an aspirating language, is unexpected if the featural make up of the segments differs in English and Russian. Therefore, accepting the feature marking hypothesis of laryngeal realism and the link between MMN asymmetries and feature marking proposed by Eulitz and Lahiri (2004) and others (e.g., Scharinger et al., 2010, 2012; Cornell et al., 2011, 2013; de Jonge and Boersma, 2015; Schluter et al., 2016), a possible conclusion is that a feature such as [SPREAD GLOTTIS] (or any other that marks the voiceless consonants instead of the voiced ones) is uniformly active for Russian stops and fricatives. Alternatively, under a relatively phonetically abstract, binary voicing feature account, there may be a cross-linguistically universal marking of [-VOICE].

## General Discussion

A recurrent pattern in all the data presented here is an asymmetric MMN response in which the voiceless segments give rise to smaller *deviant-minus-standard MMNs* than voiced ones. According to the link between MMN asymmetries and feature marking proposed by Eulitz and Lahiri (2004) and others (e.g., Scharinger et al., 2010, 2012; Cornell et al., 2011, 2013; de Jonge and Boersma, 2015; Schluter et al., 2016), this pattern could indicate that voiceless segments are the marked ones and the voiced ones are unmarked. This data pattern, and suggested theoretical account, appears to hold across English, Arabic, and Russian.

These results run counter to asymmetries observed in phonetic studies and acquisition data which have been taken to support laryngeal realism (e.g., Kager et al., 2007; Beckman et al., 2011). Particularly interesting is the data from Russian stops, which shows the same pattern as the Russian, English, and Arabic fricatives and English stops. One might expect that fricatives would pattern together, but the stops clearly use the cue of pre-voicing and short-lag aspiration in which the specification of [VOICE] as the active feature is most expected. These results strongly suggest that a voice-voiceless contrast, regardless of the phonetic implementation (i.e., voiced against plain or aspirated against plain) is encoded in the same way across languages, which favors a view of distinctive features in which they abstract away from considerable articulatory-phonetic detail. Furthermore, we suggest that this feature is not [VOICE] as commonly thought since Chomsky and Halle (1968), but [SPREAD GLOTTIS], or any other feature account where the voiceless consonant series are marked for their laryngeal articulators.

### Do the MMN Results Reflect Phonological Structure?

The strong theoretical conclusion above has to be tempered by a discussion about how much trust can be put into the assumption that the MMN methodology employed in this study actually reveals anything about long-term phonological representations as opposed to simply phonetic or acoustic representations. Even though each token used in these studies was a bare fricative (i.e., [s], [z]), with the exception of the Russian stops (i.e., [te] and [de]), 5–10 distinct tokens of each type were used in these experiments. This was a deliberate attempt to include enough inter-token variation to induce or facilitate access to long-term phonological memory representations.^14^ This design choice proved successful in the MEG study by Kazanina et al. (2006), where phonology-specific MMF results were observed: Russian (which has a voicing contrast) showed an MMF for stop consonants whereas Korean (where stop voicing is allophonic) showed no MMF response. Our use of a variety of recorded tokens (Experiment 1: 5 tokens of each type, Experiment 2: 6 tokens of each type, Experiment 3: 10 tokens of each type) to tap into language-specific representations (cf. Phillips et al., 2000; Hestvik and Durvasula, 2016) has also been apparently successful in demonstrating phonology-specific differences in Mandarin Chinese speakers compared to naïve speakers when investigating the marking of tonal contrasts (Politzer-Ahles et al., 2016). Furthermore, the results of Truckenbrodt et al. (2014) suggest that morphophonological representations related to voicing (i.e., German final devoicing) can be tapped into by the MMN technique. Finally, the fact that we were investigating cross-linguistic data that is distinct in its acoustic and phonetic details to begin with (if voicing is articulatorily and acoustically encoded in different ways across languages) is also an argument that can be used against positing an acoustic-phonetic locus for our results.

Another possible limitation to our conclusions may stem from the fact that our participants were, for the most part, bilinguals. Our native Arabic and Russian speaking subjects, by necessity, are all late bilinguals (having learned English at school), but in an English-dominant university environment. Therefore, the fact that we find MMN patterns that are English-like across the different language samples could be, in principle, just a reflection of all the participants having an English phonological grammar that our study could be tapping into. However, despite this possibility, we do not think this potential alternative explanation is very likely for the following reasons: First, during the study they were processing their native language (in the form of reading subtitles in Arabic or Russian) during the entirety of the recording. Second, the results of Experiment 2 for the interdentals does reflect a certain amount of language-specificity, as the non-native contrast did not show a clear asymmetrical MMN effect in the Arabic speaking group, and the Arabic interdental contrast did not show the same effect in the English speaking group. Therefore, if bilingualism with English did affect our results, it did not seem to have had an across-the-board effect. Third, in the Russian experiments, we included fricative contrasts that do not exist in English (like [ȿ] vs. [ʐ]), and that are therefore unlikely to be affected by the late acquisition of English phonology. Finally, while we cannot entirely rule out the influence of English or the English-speaking environment in our findings, it is important to note that the implication that late bilingualism with English could completely explain away our results would be rather drastic. Namely, it would mean that a second language phonology can completely override one’s native phonology. Crucially, this implication is incompatible with bilingual studies showing the remarkable resilience of one’s native phonological system even in early and extremely fluent bilinguals (e.g., Pallier et al., 2001).

A potential alternative explanation a reviewer raised for the pattern of results reported here is that they could be due to language-general perceptual processes, rather than to shared phonological representations across languages. Specifically, across all three experiments, larger MMNs were obtained when going from a voiceless standard to a voiced deviant, and smaller MMNs when going from a voiced standard to a voiceless deviant. Importantly, voiced segments tend to have a broader frequency spectrum (possessing energy across a wider range of frequencies), whereas voiceless segments tend to have a narrower spectrum. Thus, is it possible that the results reflect perceptual differences in moving from a broad spectrum to a narrow spectrum or vice versa, rather than phonological underspecification? This is particularly relevant since the present study used a relatively short inter-stimulus interval, which may make the observed cortical responses more likely to reflect lower-level perceptual processes as opposed to higher-level phonological processes (May and Tiitinen, 2010). Whether such an effect exists is an empirical question that may warrant further investigation (we are not aware of previous studies investigating the role of spectral width in non-linguistic stimuli). However, some existing research suggests that the shape of the spectrum may actually have the opposite effect. Specifically, many behavioral studies have shown that across languages, people are better at perceiving shifts from central to peripheral vowels, compared to shifts from peripheral to central vowels (Polka and Bohn, 2003, 2011). It has been argued that one reason for this is that peripheral vowels exhibit more formant convergence, with their spectral peaks are closer together (Schwartz et al., 2005). In other words, there is some evidence that moving from a broad to a focal spectrum should elicit greater discrimination accuracy (and, by extension, probably greater MMN amplitude) than moving from a focal to a broad spectrum. This is the opposite of what we observed in the present study (greater MMN amplitude when moving from voiceless, narrower-spectrum stimuli to voiced, broader-spectrum stimuli). It is an open question whether these patterns observed in vowels would apply to fricatives and stops, but we must leave this for future investigation. For the present purposes, however, we consider that these patterns constitute evidence that, based on the best predictions available at the moment, attributing our results to phonological underspecification provides a better account for the present findings than attributing them to acoustic/perceptual factors.

One additional concern is the degree to which features are primarily articulatory in nature rather than acoustic (cf. e.g., Halle, 2005). While there must be some link between speech perception and speech production, it remains a possibility that the disassociation between acoustics and articulation remains a confound when investigating featural representations. It may be possible that our perception study is not tapping into the same kinds of features that a production study might. The fact that our Russian [te] and [de] tokens evoked the same pattern of responses as all the fricatives—as well as the acoustically distinct English [t^h^a] and [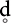
a] tokens of Hestvik and Durvasula (2016)—suggests that this may not be a simple effect of acoustics, however, as the short-lag VOT segments ([t] or [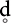]) seems to pattern differently in English and Russian.

### Feature Valuation

Assuming that our MMN results do shed light onto long term phonological structure of distinctive features, and that these results support a relatively abstract feature account, what can be concluded about its valuation? Some theories of phonology suggest that [-VOICE] or [SPREAD GLOTTIS] is universal or that binary [±VOICE] is necessary due to the sound patterning in specific languages. Vaux (1998) suggests that voiceless fricatives are universally specified as [SPREAD GLOTTIS]. Our results here are completely in line with such a claim, but the evidence from Russian stops further suggests that all voiced-voiceless contrasts in obstruents are featurally the same. Other theorists have suggested the need for [VOICE] as a binary feature and for [-VOICE] (i.e., voiceless unaspirated segments) to be phonologically active. Wetzels and Mascaró (2001) suggest that the facts of Ya:thê (Macro-Jê) argue the need for the voiceless unaspirated stops to have an active feature as both /t/ and /t^h^/ condition devoicing of a previous voiced obstruent. Similarly, Bennett and Rose (unpublished) show that the Thetogovela dialect of Moro (Kordofanian; Niger-Congo) contrasts pre-voiced stops with short-lag stops yet shows [-VOICE] to be the key feature explaining dissimilation. All of these results are compatible with our findings.

While it has been assumed since Chomsky and Halle (1968) that voicing (e.g., [+VOICE]) is the default for vowels and sonorants (but not obstruents which are considered to be voiceless by default) the evidence here suggests that voicing is, rather, the default for all obstruents and voicelessness is marked. Indeed, Chomsky and Halle themselves suggest that the default state of the vocal tract is ideal for voicing and that spread vocal folds require the most gestural effort, suggesting they could have easily called their voice feature [SPREAD GLOTTIS] if they wished it grounded in articulation (p. 300–301, 327). Our results here suggest a very similar account.

## Conclusion

In a series of three experiments, we tested the different predictions of two types of models of how distinctive laryngeal features are organized in long-term phonological memory using an MMN paradigm that has been interpreted in many studies as providing insight into the precise structure of phonological representations. The first type of model posits that two-way laryngeal contrasts are represented in long-term memory in a format that abstracts away from their precise articulatory details. The second type of model posits that the long-term memory representation of two-way laryngeal contrasts is closely related to their precise articulatory details. Across three experiments and a variety of segments, we found MMN asymmetries in the same direction for English, Arabic, and Russian, despite the putatively different phonological specification of the voiced-voiceless contrast in these languages. This consistent pattern of results is incompatible with the feature marking hypothesis espoused by laryngeal realism. Furthermore, these results are also somewhat incompatible with traditional single-feature models (e.g., Chomsky and Halle, 1968) where [+VOICE] is taken as the marked value. However, the observed results are compatible with binary or privative feature models if one posits that voicelessness—e.g., [-VOICE] or [SPREAD GLOTTIS]—is the fully specified or marked feature (e.g., Vaux, 1998; Wetzels and Mascaró, 2001; Bennet and Rose, unpublished).

## Ethics Statement

This study was carried out in accordance with the recommendations of NYUAD Procedures for Human Subjects Research Protection, NYUAD Institutional Research Board and the UAEU Ethics Committee with written informed consent from all subjects. All subjects gave written informed consent in accordance with the Declaration of Helsinki.

## Author Contributions

KS, SP-A, and DA designed the experiments. KS and SP-A implemented the experiments. KS collected the data. KS and SP-A analyzed the data. KS, SP-A, and DA discussed the analyses, the results and their interpretation. MAK and DA provided resources and the research facilities for the project. KS, SP-A, MAK, and DA wrote the manuscript.

## Conflict of Interest Statement

The authors declare that the research was conducted in the absence of any commercial or financial relationships that could be construed as a potential conflict of interest.

## Footnotes

1 Exemplar models (e.g., Goldinger, 1996, 1998; Johnson, 1997; Pierrehumbert, 2001, among others) deny the notion of the phoneme and features.

2 Following the International Phonetic Alphabet, the superscript *h* indicates aspiration, while ring below indicates a fully or partially voiceless sound. We distinguish [] from [t] only to graphically clarify the two-way contrast between aspirating and voicing languages (see **Figures 1**, **2**).

3 Voiceless sonorants are relatively rare in the languages of the world but do exist as well; a third type of obstruent, affricates, combine properties of stops and fricatives (Ladefoged, 1975).

4 Some languages show other patterns of fricative contrasts. Burmese, for example, contrasts aspirated fricatives with unaspirated and voiced ones (i.e., /s^h^/ vs. /s/ vs. /z/), while Korean has a distinction between plain and tense fricatives (i.e., /s/ and /[scale=.50]img001/).

5 Other languages include other phonation types, such as Hindi and Urdu which have a category analyzed as both voiced and aspirated, also known as murmured voice. Language like Georgian or Amharic use ejective consonants to make a three-way contrast in yet another way. See Edmondson and Esling (2006) for other types of laryngeal states used in language.

6 Exemplar models of phonology (e.g., Goldinger, 1996, 1998; Johnson, 1997; Pierrehumbert, 2001), of course, do the opposite and focus on the fine phonetic details of each exemplar.

7 Though not their terminology: we equate their *fully specified* with *marked* and *underspecified* with *unmarked* in the sense of overtly coded or specified (cf. Haspelmath, 2006). The difference is subtle and orthogonal to our purpose here, but underspecified features are argued to be absent at a highly abstract level (i.e., phonology) but must be eventually present as a default value at a lower level of representation (i.e., phonetics, acoustics, or articulation) otherwise a segment underspecified for any feature (e.g., place, manner, or voicing) would be unpronounceable. However, it should also be noted that the notion of underspecification is not universally accepted and is in and of itself a topic of debate in terms of linguistic theory (Mester and Itô, 1989; McCarthy and Taub, 1992) and psycholinguistics (Gow, 2001, 2002, 2003; Gaskell, 2003; Mitterer and Blomert, 2003; Mitterer, 2011; Ren and Morgan, 2012). Nonetheless, because it is a mechanism that has allowed some researchers (Lahiri and Reetz, 2002, 2010; Eulitz and Lahiri, 2004, a.o.) to make and test the concrete predictions about phonological organization of speech categories using electrophysiological methods, we tentatively assume underspecification to hold for the purpose of our studies.

8 Durvasula et al. (2015) also tested this prediction for tokens of [sa] and [za] and their preliminary results are compatible with ours. They also investigated the effects of using a single token to avoid access to phonological representations, but not cross-linguistic data to test the predictions of laryngeal realism.

9 The Arabic data collection was primarily conducted at the UEAU campus, which is gender-segregated. For this reason and because of other cultural norms, it was easier to recruit female participants. We know of no research suggesting the MMN should be moderated by participant gender.

10 An alternative way to conduct these comparisons is to code the token language as native vs. non-native, rather than as English vs. Arabic (e.g., rather than comparing English-speaking listeners’ asymmetry on English dentals to Arabic-speaking listeners’ asymmetry on English dentals, we can compare English-speaking listeners’ asymmetry on their own language’s [English] dentals to Arabic-speaking listeners’ asymmetry on their own language’s [Arabic] dentals). In this analysis the pattern of results is overall the same as that described above. No negative-going differences are found for native dentals (no clusters), non-native dentals (*p*s > 0.316), or native interdentals (*p*s > 0.330), but a marginal difference is found in non-native interdentals (*p* = 0.075, based on a 114-143 ms cluster). Significant positive differences (i.e., in the direction not predicted by laryngeal realism) are found for native dentals (*p* < 0.001), non-native dentals (*p* < 0.001), and non-native interdentals (*p* = 0.020, based on a 199-276 ms cluster), although not for native interdentals (no clusters).

11 The vicissitudes of history have reduced a four-way coronal stop contrast (/t/, /d/, /t^ʕ^/, and /dʕ/) in classical Arabic to only three in Emirati Arabic as the older stop /dʕ/ merged with the fricative /ðʕ/. The coronal fricatives likewise have three-way distinctions now: /s/, /z/, and /sʕ/ as well as /θ/, /ð/, and /ðʕ/. Thus, the link between voicelessness and pharyngealization is unclear phonologically.

12 One may expect different results from a language such as Thai or Swedish where the [VOICE] and [SPREAD GLOTTIS] are both needed but cannot occur in the same segment compared to a language like Hindi where one class of stops are argued to be both voiced and aspirated (carrying the necessary features) at the same time.

13 Both [ȿ] and [ʐ] represent retroflex consonants, voiceless and voiced, respectively.

14 One reviewer points out that this may be necessary, but not sufficient, to induce phonological effects.
