# Supplementary material for: Laryngeal Features Are Phonetically Abstract: Mismatch Negativity Evidence from Arabic, English, and Russian
Source: Front Psychol. 2017 May 15;8:746. doi: 10.3389/fpsyg.2017.00746 (PMC5430061; doi:10.3389/fpsyg.2017.00746)
Supplement: Supplementary file 2 [file Data_Sheet_1.pdf]

Supplementary Statistics

The results of peak-picking analyses for each experiment are presented here, along with the 2×2×2×2 ANOVA for the cluster analysis of experiment 2.

Peak picking analyses selected the average negativity in a 51 ms window (25 ms before and 25ms after the most negative data point) between 100 and 300 ms at Fz (in line with most MMN research, cf. Näätänen, 2001, Näätänen et al., 2007), with separate latencies used for each peak.

Experiment 1: Peak-picking analysis

A two-tailed paired *t*-test between deviants [v] (mean window negativity = -2.13 μV, SD = 2.51) and [f] (mean window negativity = -3.35 μV, SD = 3.0 μV) shows a significant result ($t(23)=-2.67$, mean of the differences = -1.22, 95%CI= -2.169 to -0.273, $p< 0.014$).

Experiment 2: Peak-picking analysis

The 2x2x2x2 ANOVA shows no four-way interaction ($p>.05$) but two three way interactions for both SPEAKERLANGUAGE × TOKENLANGUAGE × LARYNGEALSTATE and SPEAKERLANGUAGE × PLACEOFARTICULATION × LARYNGEALSTATE ($F(1,42)=16.2$ and $F(1,42)=9.6$ respectively, *ps*<.05 for both). There are further two-way interactions of SPEAKERLANGUAGE and LARYNGEALSTATE as well as PLACEOFARTICULATION and LARYNGEALSTATE ($F(1,42)=7.6$ and $F(1,42)=16.2$, both *ps*<.05). Furthermore this is a main effect of LARYNGEALSTATE ($F(1,42)=76.8$, $p<.05$).

Because LARYNGEALSTATE (the MMN asymmetry) and SPEAKERLANGUAGE (Eng vs Arb speakers) are of extreme interest, we divide the data along the lines of TOKENLANGUAGE: whether the speaker is listening to their native language tokens or not. In both cases we see three-way interactions still. For the incongruent language data (speakers listening to non-native tokens), there is an interaction of SPEAKERLANGUAGE × PLACEOFARTICULATION × LARYNGEALSTATE ($F(1,42)=11.5$, $p<.05$) and PLACEOFARTICULATION × LARYNGEALSTATE ($F(1,42)=36.7$), $p<.05$) as well as a main effect of LARYNGEALSTATE ($F(1,42)=38$, $p<.05$) and SPEAKERLANGUAGE ($F(1,42)=5.7$, $p<.05$). For the congruent language data (speakers listening to their own language

tokens), there is an an interaction of SPEAKERLANGUAGE × PLACEOFARTICULATION × LARYNGEALSTATE ($F(1,42)=4.5$, $p<.05$), SPEAKERLANGUAGE × LARYNGEALSTATE ($F(1,42)=6.6$, $p<.05$), and a main effect of LARYNGEALSTATE ($F(1,42)=45.5$, $p<.05$).

We then divide the both congruent and incongruent data along the lines of place (again, so that SPEAKERLANGUAGE is not divided since we're interested in knowing when the speaker is irrelevant. For speakers listening to incongruent dentals ([s] and [z]) there is an interaction of SPEAKERLANGUAGE × LARYNGEALSTATE ($F(1,42)=10.4$, $p<.05$) and a main effect of LARYNGEALSTATE ($F(1,42)=73.0$, $p<.05$) bot not SPEAKERLANGUAGE ($p>.05$). For the incongruent interdentals ([θ] and [ð]) there is an only a main effect of SPEAKERLANGUAGE ($F(1,42)=6.0$, $p<.05$). For the congruent dentals, there is an interaction of SPEAKERLANGUAGE × LARYNGEALSTATE ($F(1,42)=12.8$, $p<.05$) and a main effect of LARYNGEALSTATE ($F(1,42)=38.8$, $p<.05$). For the congruent Interdentals there is no interaction and only a main effect of LARYNGEALSTATE ($F(1,42)=19.6$, $p<.05$). Thus, there is an interaction for incongruent dentals and congruent dentals. Furthermore, we don't see a main effect for incongruent interdentals, which is consistent with the visual inspection of each condition. The consistent interactions of SPEAKERLANGUAGE and LARYNGEALSTATE suggests that the Arabic speakers have a larger asymmetry than the English speakers (though in the same direction), based on the visual inspection of the data.

For the incongruent dentals, we see an main effect of LARYNEALSTATE for both Arabic and English speakers ($F(1,21)$ =110, $p<.05$ for Arabic speakers, F(1,21=10.2 for English speakers, both ps<.05). Main effects of LARYNGEALSTATE are also found for both speaker groups for congruent dentals (F(1,21=40.8 for Arabic speakers, $F(1,21)=4.3$ for English speakers, both ps<.05).

Based on a visual inspection of the data and the ANOVA results, it is clear that the interactions are largely driven by the effect size, not the effect direction (modulo eliminating the incongruent speaker-token data). The direction of the asymmetries for subject's native-language tokens are all the same. The size of the MMNs vary, which indicates a difference between the speaker groups which may be language driven. Furthermore, the results of the incongruent interdentals show differences between English and Arabic speakers as well.

The results of individual two-tailed paired t-tests for each contrast (Holm-Bonferroni corrected for multiple comparisons) are shown below in Table 1. The direction of the asymmetries for subject's native-language tokens are all the same. The size of the MMNs vary, which indicates a difference between the speaker groups which may be language driven. Furthermore, the results of the incongruent interdentals show differences between English and Arabic speakers as well.

	**Token Language**	**Place of Articulation**	**Mean Difference**	***t*(23)**	**95% CI**	***p***	**Holm-corrected *p***	
**English**	Native	sz	1.21	2.39	0.16–2.25	0.025	0.076	?
	Native	θð	1.84	3.80	0.84–2.85	0.001	0.006	*
	Non-Native	sz	1.90	3.34	0.72–3.07	0.003	0.014	*
	Non-Native	θð	0.60	1.46	-0.25–1.44	0.003	0.319	
**Arabic**	Native	sz	4.35	7.12	3.09–5.61	0.159	0.000	*
	Native	θð	2.28	3.02	0.72–3.85	0.000	0.024	*
	Non-Native	sz	4.35	9.74	3.42–5.27	0.006	<0.001	*
	Non-Native	θð	-0.08	-0.14	-1.24–1.08	<0.001	0.886	

Table 1 – Conditions and statistics for planned comparisons, including Holm-Bonferroni adjusted *p*-values.

Experiment 2: Repeated Measures ANOVA for Cluster Analysis

The crucial effect of interest in this experiment was the interaction between deviant LARYNGEALSTATE (voiced or voiceless; this effect represents the MMN asymmetry) and LISTENERLANGUAGE, as such an interaction would indicate different asymmetries for English and Arabic speakers. This effect was significant ($p = .011$), based on a cluster of samples from 100 to 165 ms and including 26 channels: Fp1, Fp2, F3, Fz, F4, F8, FC5, FC1, FC2, FC6, C3, Cz, C4, T8, CP5, CP1, CP2, CP6, P3, Pz, P4, P8, O1, Oz, O2, and FCz. In this cluster, there was a stronger asymmetry (i.e., the extent to which voiced deviants elicited more negative MMNs than voiceless deviants was greater) for Arabic than for English speakers. If anything, this is interaction is in the opposite direction as the initial prediction (which was that Arabic speakers would have a more negative MMN for voiceless deviants whereas English speakers would have a more negative MMN for voiced deviants). Although it was qualified by the abovementioned interaction, there was also a significant main effect of LARYNGEALSTATE, based on a cluster from 100–224 ms and including all channels, in

which voiced deviants elicited more negative MMNs than voiceless deviants. This effect was qualified by numerous relevant interactions, including LARYNGEALSTATE × PLACEOFARTICULATION, LARYNGEALSTATE × TOKENLANGUAGE, LARYNGEALSTATE × PLACEOFARTICULATION × LISTENERLANGUAGE, LARYNGEALSTATE × TOKENLANGUAGE × LISTENERLANGUAGE, and LARYNGEALSTATE × PLACEOFARTICULATION × TOKENLANGUAGE × LISTENERLANGUAGE (as well as the abovementioned LARYNGEALSTATE × LISTENERLANGUAGE interaction). All these interactions, however, were due to differences in the magnitude of the asymmetry, rather than differences in the direction of the asymmetry; as shown in (figure 6), every condition—except the non-native interdentals—elicited a significant asymmetry in the same direction, and for all but English interdental fricatives the asymmetry was larger for Arabic listeners than for English listeners, contra our prediction. Thus, since none of these more complex interactions is indicative of a difference we can attribute to phonology, we do not break them down further here.

Experiment 3: Peak-picking analysis

In a 3x2 ANOVA—CONTRAST (SZ, Fricatives, TeDe) × DEVIANT (Voiced, Voiceless) there is no interaction of the two factors ($F(2,42)=1.80$, $p=.176$) and a main effect of the chosen contrast ($F(2,42)=12.6$, $p<.001$) and DEVIANT ($F(1,23)=43.5$, $p<.001$).
